# Dietary intake and food sources of one-carbon metabolism nutrients in preschool aged children

**DOI:** 10.1038/s41430-018-0376-7

**Published:** 2018-12-11

**Authors:** Rachael M. Taylor, Roger Smith, Clare E. Collins, Tiffany-Jane Evans, Alexis J. Hure

**Affiliations:** 1 Priority Research Centre for Reproductive Science, University of Newcastle, Callaghan, NSW 2308 Australia; 2 Faculty of Health and Medicine, School of Medicine and Public Health, University of Newcastle, Callaghan, NSW 2308 Australia; 3 Hunter Medical Research Institute, 1 Kookaburra Circuit, New Lambton Heights, NSW 2305 Australia; 4 Faculty of Health and Medicine, School of Health Sciences, University of Newcastle, Callaghan, NSW 2308 Australia; 5 Priority Research Centre in Physical Activity and Nutrition, University of Newcastle, Callaghan, NSW 2308 Australia; 6 Clinical Research Design IT and Statistical Support (CReDITSS) Unit, Hunter Medical Research Institute, 1 Kookaburra Circuit, New Lambton Heights, NSW 2305 Australia; 7 Priority Research Centre for Generational, Health and Ageing, University of Newcastle, Callaghan, NSW 2308 Australia

**Keywords:** Risk factors, Epigenetics

## Abstract

**Background:**

It is hypothesised that epigenetic mechanisms including DNA methylation may underlie the relationship between early-life nutrition and child cognitive outcomes. This study aimed to identify dietary patterns associated with the intake of one-carbon metabolism nutrients in children aged 2–3 years.

**Methods:**

A validated 120-item semi-quantitative food frequency questionnaires at 2–3 years of age were used to estimate the intake of one-carbon metabolism nutrients (methionine, folate, choline and vitamins B2, B6, B12) and to quantify mean number of serves consumed of the food groups specified by the Australian Guide to Healthy Eating (AGHE). Descriptive statistics were used to analyse the contribution of each food group and food items to the total intake of one-carbon metabolism nutrients. Linear regression was used to test for linear trends in food group servings by nutrient intake quintiles.

**Results:**

No child (*n* *=* 60) from the Women And Their Children’s Health (WATCH) study consumed the recommended number of serves for all AGHE food groups. Dairy and alternatives (18–44%), discretionary foods (6–33%) and meat and alternatives (6–31%) were the main sources of most one-carbon metabolism nutrients. Most child intakes of one-carbon metabolism nutrients exceeded the nutrient reference values (NRVs), except for the intake of choline, for which the mean intake was 9% below the adequate intake (AI).

**Conclusion:**

Dairy and alternatives, discretionary foods and meat and alternatives food groups contributed significantly to the children’s intake of one-carbon metabolism nutrients. The children generally had low intakes of meat and alternative foods, which may explain their inadequate intake of choline.

## Introduction

‘Developmental Origins of Health and Disease’ (DoHAD) is a body of research that demonstrates how early-life nutrition impacts on subsequent development and health, including neurodevelopment and the risk of developing neuropsychiatric and behavioural disorders across the lifespan [1–10]. A large (*n* *=* 13,889) cluster-randomised controlled trial reported that the duration of exclusive breastfeeding (≥3 months), after adjusting for geographical location, age at follow-up, sex, birth weight and both maternal and paternal education was strongly associated with indices of cognitive development at age 6.5 years [11]. In addition, a systematic review and meta-analysis of 16 cohort studies and one cross-sectional study, confirmed a positive association between breastfeeding and child cognition (up to the age of 15 years), with a mean difference of 2.62 points in intelligence quotient (IQ) (95% confidence interval: 1.25; 3.98) after adjusting for maternal IQ [12]. Five cohort studies (*n* *=* 22,184) have also shown that healthy complementary feeding patterns defined as a higher consumption of fruit, vegetables and home-prepared meals, as well as breastfeeding is important for cognitive function during childhood [13–17]. During the first five years of life, 70% of brain growth occurs, therefore optimal nutrition is essential during this period when the brain expresses a high level of developmental plasticity [18–20].

The mechanisms that may underlie the association between nutrition and cognition are not well understood, however epigenetic mechanisms are thought to be involved [21, 22]. Epigenetics include mechanisms that modify chromatin to generate different phenotypes, without altering the underlying DNA sequence [22–24]. Such modifications include DNA methylation, the addition of a methyl group to a cytosine nucleotide base [25]. Evidence from longitudinal analysis of the human genome indicates that DNA methylation patterns are significantly reconfigured within the first five years of life and stabilise after seven years of age [26]. DNA methylation is influenced by nutrients, with the production of methyl groups regulated by specific nutrients (folate, methionine, choline, vitamins B2, B6 and B12) within the one-carbon metabolism pathway [25]. Biochemical reactions involving these nutrients result in the production of S-adenosylmethionine (SAM), the methyl donor required for methylated DNA [27, 28]. DNA methyltransferase (DNMT) enzymes, DNMT3a and DNMT3b are responsible for transferring methyl groups to DNA which establishes new methylation patterns [29].

Evidence suggests that DNA methylation is involved in the development of cognitive abilities and behaviour via the cellular regulation of synaptogenesis and synaptic pruning [30–33]. In response to neurodevelopmental processes throughout life, synaptic transmission can undergo long-term reduction or enhancement, referred to as long-term depression and potentiation, which is essential for neural plasticity [34]. Evidence from animal models has demonstrated that brain specific-conditional deletion of DNMT1 and DNMT3a impairs neuron maturation, synaptic long-term potentiation and development of cognitive abilities [30, 35, 36].

We previously examined the relationship between postnatal one-carbon metabolism intake and global DNA methylation [37]. While no association was identified in that analysis, this could have been attributed to the limitations in analysing the impact of single nutrients on the epigenome. The effect of changes in the availability of a single nutrient involved in one-carbon metabolism on DNA methylation is likely to be small and potentially challenging to detect. Furthermore, the regulation of DNA methylation is reliant on the interaction of multiple nutrients within one-carbon metabolism, rather than the effect of a single nutrient alone [25]. However, measuring the combined effect of multiple one-carbon metabolism nutrients on DNA methylation is fraught with numerous methodological limitations, especially as the contribution of each nutrient within this metabolic pathway is not equal. It is speculated that approximately 60% of methyl groups in one-carbon metabolism are derived from choline, 20% from methionine and 10–20% from folate [38]. Therefore, a broader whole-diet approach is required in order to examine how the consumption of a combination of foods, or overall dietary patterns, contribute to the intake of one-carbon metabolism nutrients. Analysing dietary patterns will be important for understanding how interactions and synergetic or antagonistic effects of multiple nutrients impact on DNA methylation and brain function. Therefore, the aim of the current study was to evaluate the relationship between dietary patterns and the intake of one-carbon metabolism nutrients (methionine, folate, choline and vitamins B12, B2 and B6) among a sample of Australian children aged 2–3 years. The two study objectives were to: 1) Assess the contribution of usual intake of food group serves to the total intake of one-carbon metabolism nutrients; and 2) Identify foods that significantly contribute to the total intake of one-carbon metabolism nutrients.

## Methods

### Study participants

Data from the Women and Their Children’s Health (WATCH) cohort were used, which followed mother–child pairs during gestation (19, 24, 30 and 36 weeks gestation) and the postnatal period (three monthly intervals during the first year after birth and then annually until age four years) [39, 40]. Women who were less than 18 weeks pregnant, lived in the local region and able to attend scheduled study visits at John Hunter Hospital (JHH), New South Wales (NSW), Australia were eligible to participate. Pregnant women were recruited between July 2006 to December 2008 by research midwives at JHH antenatal clinic, local media coverage or word of mouth. From all the pregnant women who were invited to participate, 61% provided their consent and 181 women were enrolled in the study [40]. The majority of the WATCH sample (*n* *=* 133, 74% of sample) were retained at the two year postnatal study visit, however participants did not consistently attended all study visits [39, 41]. The WATCH study received ethics approval from the Hunter New England Research Ethics Committee (06/05/24/5.06) and all participants gave written informed consent.

### Dietary assessment

Usual dietary intakes of the WATCH children, aged two to three years (*n* *=* 60), was assessed using a food frequency questionnaires (FFQ), which has been described in detail elsewhere [41–43]. The Australian Child and Adolescent Eating Survey (ACAES) was used to assess usual dietary intake over the previous six months of children aged 2 to 17 years [44]. The ACAES has previously demonstrated acceptable accuracy for estimating energy intake in Australian children aged two to three years compared to the doubly labelled water method [44, 45]. The ACAES is a 135-item semi-quantitative FFQ with 120 food items and 15 supplementary questions including, age, nutrient supplement use, food behaviour and duration of sedentary activity. The mothers participating in the WATCH study reported their child’s consumption of specific food items over the previous six months, using frequency categories ranging from never to four or more times per day, and for some beverages up to seven or more glasses per day. Toddler-specific portion sizes were used for each food item which is derived from the 2007 National Children’s Nutrition and Physical Activity Survey [46]. An Accredited Practising Dietitian (APD) checked all questionnaires for completeness and the mothers were contacted for clarification or completion of missing questions.

### Dietary analysis

#### Quantifying folate, vitamins B2, B6 and B12 intake

To compute the child’s intake of one-carbon metabolism nutrients (methionine, folate, vitamins B2, B6 and B12) the FFQ raw data was analysed using the software, Foodworks 7, 2012 (Xyris Software (Australia) Pty Ltd, Brisbane, Queensland) which includes the current food composition data used in Australia (AUSNUT 2013 [47], AUSNUT 2007 [48], NUTTAB 2010 [49]). The Australian Nutrition Tables (NUTTAB 2010) nutrient composition data for each food item is primarily (74%) derived from Australian laboratory food analysis. However, the availability of composition data for specific nutrients, especially for vitamins B6 and B12, varies between food items [49]. The AUSNUT 2007 was also used which estimates the nutrient composition of 4225 foods, beverages and dietary supplements consumed by children aged 2–16 years in the 2007 National Children’s Nutrition and Physical Activity Survey [48]. However, this database does not provide food composition values for vitamins B12 and B6 [48]. Therefore, to obtain additional food composition data for vitamins B6 and B12, AUSNUT 2013 was used which provides data based on food and beverages consumed from the 2011–3 Australian Health Survey [47].

#### Quantifying methionine intake

Child dietary intake of the amino acid methionine, was first quantified from the FFQs using the NUTTAB 2010 amino acids database [50]. The methionine content of 16 FFQ food items was quantified using Australian food composition data, 94 FFQ food items were quantified using data obtained from the United States Department of Agriculture (USDA) National Nutrient Database for Standard Reference, release 28 (2016) [51]. The remaining 10 food items did not contain any methionine.

#### Quantifying choline intake

Currently, there is no Australian food composition data available for the nutrient, choline. Therefore the USDA database for the choline content of common foods, release 2, 2008 [52] and the Canadian Nutrient File (CNF) database [53] were used. The USDA database provides the choline content of 4680 food items [52], and the CNF database provides the choline of 2774 food items [53]. The choline content of 109 FFQ food items was quantified using data from the USDA, while the remaining five food items were quantified using the CNF database. The remaining six food items did not contain any choline.

#### Total FFQ intake of one-carbon metabolism nutrients

The children’s nutrient intake from the FFQ raw data were estimated by multiplying the quantity (grams) of a food item consumed per day by the nutrient content of that food item (per gram) reported by the Australian food databases for methionine, folate, vitamin B2, B6 and B12, and the USDA and Canadian databases for additional methionine and all choline. The combined total nutrient intakes from all FFQ food items were used to estimate a child’s daily intake of specific one-carbon metabolism nutrients.

#### Quantifying food group serves

The raw child FFQ data were categorised into the food groups, as defined in the Australia’s national food selection guide, the Australian Guide to Healthy Eating (AGHE) (Table 1) [54]. These food groups include grains (including breads and cereals), vegetables, fruit, dairy products/dairy alternatives (including non-dairy milk varieties) and meat/non-meat alternatives (including legumes, lentils, nuts and eggs) [54]. Discretionary food items include those that do not belong to any of the five nutrient-dense, core food groups and tend to be energy-dense and nutrient-poor contribution, hence the reason that limited consumption is recommended [54]. The mean daily number of servings per food group was estimated using the FFQ data and the serving sizes specified within the AGHE (Table 1) [54]. The serving size of food items not specified in the AGHE, were derived based on food items with a comparable energy content within the same AGHE food group. For example, a standard serving of cheese spread/cream cheese was deemed to be 55 g which is equivalent to the energy content of 250 mL of plain milk, 40 g of hard cheese and 120 g ricotta cheese. To determine the number of serves consumed for each food group contained within a mixed food dish, the ingredients were disaggregated from the food dish and allocated to each food group. For example, the FFQ item ‘beef/lamb pieces with vegetables’ was disaggregated into meat and vegetable food group serving based on a weight ratio of 2:1.

**Table 1:** Food frequency food items included in the Australian Guide to Healthy Eating food groups

AGHE food group	Single serving size	FFQ food items included
Grains	30 g	Muesli or breakfast cereal
	120 g	Cooked porridge
	40 g	Bread, pita, roll, English muffin, bagel or crumpet
	100 g	Rice, noodles or pasta
	35 g	Savoury biscuits, crispbread or crackers
Vegetables	75 g	Potato, pumpkin, sweet potato, cauliflower, green beans, spinach, cabbage, brussel sprouts, peas, broccoli, carrots, zucchini, eggplant, squash, capsicum, corn, mushrooms, tomatoes, lettuce, celery, cucumber, onion, spring onion or leek
	30 g	Avocado
Fruit	150 g	Canned fruit, fruit salad, apple, pear, orange, mandarin, grapefruit, banana, peach, nectarine, plum, apricot, mango, paw paw, pineapple, grapes, strawberries, blueberries, melon
	30 g	Dried fruit
	125 mL	Fruit juice drink
Dairy and alternatives	250 mL	Full-cream, reduced fat or skim cow’s milk (plain or flavoured)
	250 mL	Soy or rice milk (plain or flavoured)
	200 g	Yoghurt
	40 g	Cheese
	150 g	Cottage or ricotta cheese
	55 g	Cheese spread or cream cheese
Meat and alternatives	65 g	Beef, lamb, pork, liver or mince
	80 g	Chicken
	100 g	Fresh or canned seafood
	120 g	Eggs
	170 g	Soybeans or tofu
	150 g	Lentils, baked beans or chickpeas
	40 g	Nuts
Discretionary foods	600KJ	Soft drinks, fruit juice, cordial, cream, ice cream, frozen yoghurt, cakes, muffins, scones, sweet pastries, desserts, sweet biscuits, combination snacks, snack bars, snack noodles, soup, tacos, burritos, enchiladas, sausages, frankfurts, pluto pup, hamburgers, pizza, savoury pastries, hot dog, hash brown, potato scallops, potato chips (crisps), chips (other flavours) ice blocks, chocolate, lollies, salad dressing, jam, honey, golden syrup, marmalade, peanut butter, Nutella, vegemite (yeast spread), sauces, Devon (luncheon meat), salami, bacon, ham, jelly, french fries, crumbed chicken or fish

### Statistical analysis

Descriptive statistics were used to summarise maternal and child characteristics, children’s daily consumption of the AGHE food groups, one-carbon metabolism nutrient intakes and food group contributions to these nutrient intakes at all time points. The demographic characteristics of the included WATCH mothers (*n* *=* 59) were compared to the remaining mothers (*n* *=* 122) not included in the current analysis using Fisher’s extact tests. A two sample t-test was used to compare maternal age of both groups. Differences between the WATCH children’s estimated daily consumption of the AGHE food groups compared to the recommended daily AGHE food group serves were assessed using one-sample t-tests (two-tailed *p* values reported). Comparisons were made for the WATCH children at two and three years of age, as well as the combined population (averaging repeated measures over the two time points). The percentage of contribution for each food group to the intake of one-carbon metabolism nutrients was estimated by summing the total nutrient intake of a food group for all children and dividing this value by the sum of the nutrient from all food items and children. For each one-carbon metabolism nutrient, the children’s nutrient intake was ranked into quintiles, where higher quintile represented higher intake. In addition, the average number of serves for each food group was calculated for each quintile. Linear tests for trend were conducted using linear regression for nutrient quintiles by food group serves. Multiple testing was accounted for using the Benjamini and Hochberg False Discovery Rate (FDR) method [55, 56]. Eighty-nine tests were conducted and the adjusted p-value threshold was 0.0197. All statistical analyses were performed using the Statistical Analysis System (SAS) software (version 9.4) (SAS Institute, Cary, North Carolina, USA).

## Results

### Sample characteristics

The subset of children (*n* *=* 60) analysed from the WATCH cohort had a median age of 2.5 years and 52% were girls. The median age of their mothers at delivery was 30.6 years and the majority were married (65%) and had attained at least a year-12 educational qualification (50%). No significant difference (*p* < 0.0197) was found between the maternal characteristics of the WATCH mothers included (*n* *=* 59) in the current analysis compared to the mothers not included (*n* *=* 122) in the current study.

### Consumption of the AGHE food groups

The number of serves recommended for consumption of the AGHE food groups for children aged 2–3 years is summarised in Table 2. In addition, the mean number of serves consumed of the AGHE food groups by the WATCH children, as well as the percentage of children who consumed the AGHE recommended number of daily serves of each food group. None of the WATCH children at age 2–3 years consumed the recommended daily number of serves for all the food groups. The mean number of serves consumed per food group was significantly different compared to the AGHE recommendations for all food groups for the combined age groups (all *p* values < 0.0197). However, at two and three years of age, the mean consumption of fruit (excluding juice) was not significantly different from the AGHE recommendations (*p* = 0.02). In addition, at three years of age, the consumption of dairy and alternatives was not significantly different from the AGHE recommendations (*p* = 0.03). For the combined ages, 80% of children consumed one or more servings of discretionary foods per day. The mean consumption of discretionary foods at two years of age was two serves, which increased to 2.7 serves at three years of age.

**Table 2:** Consumption of the Australian Guide to Healthy Eating food groups in children (*n* *=* 60) aged two and three years in the Women and Their Children’s Health (WATCH) Study

		Daily mean servings per day ± SD			Proportion meeting the recommended daily servings, n (%)		
AGHE food group	Daily recommended servings	2 years *n* *=* 40	3 years *n* *=* 30	Combined *n* *=* 60	2 years *n* *=* 40	3 years *n* *=* 30	Combined *n* *=* 60
Grains	4	1.9 ± 0.7*	1.8 ± 0.7*	1.9 ± 0.7*	0 (0)	0 (0)	0 (0)
Vegetables	2.5	1.4 ± 1.0*	1.3 ± 0.8*	1.4 ± 0.9*	5 (13)	1 (3)	6 (9)
Fruit (excl. juice)	1	1.3 ± 0.8	1.6 ± 1.2	1.4 ± 1.0*	24 (60)	20 (67)	44 (63)
Fruit (incl. juice)	1	1.8 ± 0.9*	2.1 ± 1.3*	1.9 ± 1.1*	34 (85)	25 (87)	59 (84)
Dairy and alternatives	1.5	2.3 ± 1.1*	2.2 ± 1.7	2.2 ± 1.4*	29 (73)	20 (67)	49 (70)
Meat and alternatives	1	0.7 ± 0.3*	0.8 ± 0.4*	0.7 ± 0.3*	4 (10)	8 (27)	12 (17)
Discretionary foods	0–1	2.0 ± 1.1*	2.7 ± 1.9*	2.3 ± 1.5*	6 (15)	6 (20)	12 (17)

Statistically significant difference compared with the AGHE recommended servings per day: * *p* *<* 0.0197

*AGHE* Australian Guide to Healthy Eating, *excl* excluding; incl, including; n, number of children, *SD* standard deviation

### Contribution of AGHE food groups to one-carbon metabolism nutrient intake

#### Methionine

The estimated mean total intake of methionine per day was 922 mg (±285 mg) at two years of age and 901 mg (±301 mg) at three years of age. In Australia, no nutrient reference values (NRVs) have been set for specific amino acids, however the Food and Nutrition Board: Institute of Medicine recommends that 5–20% of energy intake should be consumed from protein for children aged 1–3 years [57]. Previously findings from the WATCH cohort reported that 89% of children met this recommendation at 2–3 years of age [43]. The main food group sources of methionine intake for the WATCH children in the combined age groups were dairy and alternatives (45%), meat and alternatives (23%) and discretionary foods (16%) (Table 3). Using simple linear regression, as the quintiles of methionine intake at two years of age increased (*p* < 0.0197) the daily mean number of serves consumed of grains, dairy and alternatives and discretionary foods were also greater (Table 4). As the quintiles of methionine intake at three years of age increased, the daily mean number of serves consumed of dairy and alternatives and meat and alternatives were also greater (*p* < 0.0197) (Table 5). The top five ranked foods for contributing to methionine intake that occurred at both two and three years of age were milk, cheese, yoghurt (not frozen) and meat mince (Table 6).

**Table 3:** Mean intake of one-carbon metabolism nutrients and the overall percentage of contribution of each food group to one-carbon metabolism intake among WATCH children

	Mean daily methionine nutrient intake (mg)±SD	% Total nutrient intake				
Food group	2 years (*n* *=* 40)	3 years (*n=*30)	Combined (*n=*60)	2 years (*n=*40)	3 years (*n=*30)	Combined *(n=*60)
Grains	100.58 ± 44.14	94.66 ± 35.54	98.04 ± 40.51	10.91	10.50	10.74
Vegetables	34.69 ± 21.17	32.91 ± 18.18	33.93 ± 19.82	3.76	3.65	3.72
Fruit (excl. juice)	19.92 ± 13.06	20.31 ± 16.45	20.09 ± 14.50	2.16	2.25	2.20
Fruit (incl. juice)	21.12 ± 12.70	21.65 ± 16.47	21.35 ± 14.32	2.29	2.40	2.34
Dairy and alternatives	452.98 ± 215.38	343.67 ± 248.86	406.13 ± 234.98	49.15	38.12	44.48
Meat and alternatives	178.66 ± 87.61	249.13 ± 144.25	208.86 ± 119.65	19.39	27.64	22.88
Discretionary foods	134.80 ± 78.99	160.81 ± 114.71	145.94 ± 96.05	14.63	17.84	15.99
Grains	63.30 ± 28.89	55.50 ± 29.69	59.95 ± 29.28	33.23	28.59	31.36
Vegetables	25.15 ± 16.66	23.37 ± 14.68	24.39 ± 15.76	13.57	12.04	12.76
Fruit (excl. juice)	15.96 ± 10.71	17.41 ± 14.63	16.58 ± 12.46	8.43	8.97	8.67
Fruit (incl. juice)	18.33 ± 10.32	19.83 ± 14.93	18.98 ± 12.43	9.70	10.21	9.93
Dairy and alternatives	35.93 ± 15.00	30.26 ± 16.36	33.50 ± 5.74	18.65	15.59	17.52
Meat and alternatives	9.87 ± 6.26	14.44 ± 12.37	11.83 ± 9.58	5.24	7.44	6.19
Discretionary foods	44.92 ± 39.72	53.17 ± 45.52	44.92 ± 39.72	20.88	27.38	23.50
Grains	15.91 ± 6.77	14.47 ± 5.37	15.29 ± 6.21	8.65	8.00	8.38
Vegetables	14.87 ± 9.61	13.89 ± 8.11	14.45 ± 8.95	8.08	7.69	7.92
Fruit (excl. juice)	12.40 ± 7.84	12.89 ± 10.42	12.61 ± 8.97	6.74	7.13	6.91
Fruit (incl. juice)	13.48 ± 7.61	14.10 ± 10.49	13.75 ± 8.89	7.33	7.80	7.53
Dairy and alternatives	66.28 ± 37.10	52.85 ± 36.55	60.53 ± 37.21	36.05	29.24	33.16
Meat and alternatives	33.63 ± 21.63	45.61 ± 32.23	38.76 ± 27.14	18.29	25.24	21.24
Discretionary foods	40.78 ± 36.32	41.02 ± 22.99	40.88 ± 31.11	22.18	22.70	22.40
Grains	0.33 ± 0.16	0.29 ± 0.19	0.31 ± 0.17	16.11	12.78	14.60
Vegetables	0.09 ± 0.07	0.08 ± 0.05	0.09 ± 0.06	4.58	3.59	4.13
Fruit (excl. juice)	0.10 ± 0.06	0.09 ± 0.08	0.10 ± 0.072	4.80	4.11	4.49
Fruit (incl. juice)	0.10 ± 0.06	0.10 ± 0.08	0.10 ± 0.07	4.80	4.23	4.55
Dairy and alternatives	0.88 ± 0.49	0.70 ± 0.52	0.80 ± 0.51	42.91	31.07	37.55
Meat and alternatives	0.11 ± 0.06	0.17 ± 0.18	0.13 ± 0.13	5.25	7.51	6.27
Discretionary foods	0.54 ± 0.59	0.93 ± 1.11	0.71 ± 0.87	26.36	40.94	32.96
Grains	0.10 ± 0.04	0.12 ± 0.16	0.10 ± 0.11	12.49	10.59	11.60
Vegetables	0.08 ± 0.05	0.08 ± 0.08	0.08 ± 0.07	9.90	7.59	8.70
Fruit (excl. juice)	0.16 ± 0.11	0.15 ± 0.15	0.16 ± 0.13	20.99	14.20	17.49
Fruit (incl. juice)	0.17 ± 0.11	0.16 ± 0.14	0.17 ± 0.12	22.25	15.05	18.55
Dairy and alternatives	0.23 ± 0.16	0.24 ± 0.14	0.24 ± 0.15	30.45	22.26	26.24
Meat and alternatives	0.10 ± 0.05	0.21 ± 0.42	0.15 ± 0.28	12.74	19.60	16.27
Discretionary food	0.10 ± 0.07	0.28 ± 0.79	0.18 ± 0.52	13.43	25.61	19.70
Grains	0.08 ± 0.07	0.07 ± 0.03	0.11 ± 0.28	2.28	1.48	1.88
Vegetables	0.03 ± 0.02	0.03 ± 0.02	0.03 ± 0.02	0.82	0.58	0.70
Fruit (excl. juice)	0.00 ± 0.00	0.00 ± 0.00	0.00 ± 0.00	0.00	0.00	0.00
Fruit (incl. juice)	0.00 ± 0.00	0.00 ± 0.00	0.00 ± 0.00	0.00	0.00	0.00
Dairy and alternatives	2.45 ± 1.42	2.33 ± 1.31	2.40 ± 1.36	72.27	50.38	61.20
Meat and alternatives	0.63 ± 0.74	1.94 ± 4.11	1.23 ± 2.81	18.67	42.06	30.50
Discretionary foods	0.20 ± 0.14	0.25 ± 0.20	0.22 ± 0.17	5.96	5.49	5.73

**Table 4:** Mean food group serves per nutrient intake quintile for the WATCH children at two years of age

Food group serves	Quintiles of nutrient intake					
	0 (*n* *=* 8)	1 (*n=*8)	2 (*n=*8)	3 (*n=*8)	4 (*n=*8)	*P* value
*Methionine*						
Grains	1.56	1.68	1.61	2.42	2.26	0.0053
Vegetables	1.46	1.29	1.22	1.43	1.78	0.4729
Fruit	1.44	1.22	1.38	0.96	1.52	0.9542
Dairy and alternatives	1.28	1.45	2.4	2.63	3.49	<.0001
Meat and alternatives	0.51	0.62	0.61	0.63	0.88	0.0241
Discretionary foods	1.29	1.85	1.98	2.15	2.74	0.0062
*Choline*						
Grains	1.61	1.6	1.77	2.23	2.33	0.0067
Vegetables	1.29	0.88	1.52	1.65	1.84	0.0782
Fruit	1.39	1.97	0.99	0.78	1.45	0.2629
Dairy and alternatives	1.35	1.86	2.23	2.39	3.43	< .0001
Meat and alternatives	0.58	0.43	0.7	0.77	0.78	0.0241
Discretionary foods	1.52	1.95	2.07	2.09	2.38	0.1245
*Vitamin B6*						
Grains	1.36	1.59	2.18	1.99	2.41	0.0008
Vegetables	0.98	1.25	1.51	1.69	1.76	0.0612
Fruit	1.61	1.06	1.05	1.48	1.27	0.7684
Dairy and alternatives	1.91	1.85	1.6	2.3	3.59	0.0012
Meat and alternatives	0.46	0.53	0.6	0.9	0.76	0.0025
Discretionary foods	1.35	2.07	2.04	2.1	2.45	0.0652
*Vitamin B12*						
Grains	1.69	1.88	1.95	1.62	2.39	0.1508
Vegetables	1.71	1.22	1.19	1.95	1.13	0.6910
Fruit	1.13	1.11	1.4	1.45	1.42	0.3184
Dairy and alternatives	0.93	1.66	2.11	3.11	3.44	< .0001
Meat and alternatives	0.74	0.56	0.61	0.49	0.86	0.6336
Discretionary foods	1.92	1.57	2.56	1.57	2.39	0.4497
*Folate*						
Grains	1.2	1.84	1.87	2.14	2.48	< .0001
Vegetables	0.86	1.23	1.5	1.59	2	0.0113
Fruit	1.73	1.01	1.44	1.15	1.14	0.2215
Dairy and alternatives	2.13	1.44	1.77	2.86	3.06	0.0064
Meat and alternatives	0.47	0.57	0.63	0.87	0.73	0.0125
Discretionary foods	1.73	1.69	1.86	2.46	2.26	0.1329
*Vitamin B2*						
Grains	1.53	1.53	2.09	2.23	2.16	0.0111
Vegetables	1.22	1.22	1.39	1.69	1.66	0.2101
Fruit	1.2	1.25	1.63	1.13	1.32	0.8918
Dairy and alternatives	1.15	2.09	1.86	2.82	3.34	< .0001
Meat and alternatives	0.62	0.61	0.48	0.77	0.79	0.1329
Discretionary foods	1.92	1.77	1.38	2.11	2.82	0.0755

*P values are for linear trend across the quintiles. Statistically significant association *p* *<* 0.0197

**Table 5:** Mean food group serves per nutrient intake quintile for the WATCH children at three years of age

Food group serves	Quintiles of nutrient intake					
	0 (*n=*8)	1 (*n=*8)	2 (*n=*8)	3 (*n=*8)	4 (*n=*8)	*P* value
*Methionine*						
Grains	2.06	1.33	1.68	1.83	2.17	0.4467
Vegetables	1.01	1.14	1.62	1.48	1.31	0.3643
Fruit	0.96	1.74	2.28	1.54	1.25	0.8169
Dairy and alternatives	1.22	1.36	1.89	2.74	3.88	0.0015
Meat and alternatives	0.54	0.61	0.71	0.89	1.08	0.0065
Discretionary foods	1.33	4.02	1.98	3.01	2.94	0.3649
*Choline*						
Grains	2.07	1.18	1.52	2.12	2.18	0.2224
Vegetables	0.82	1.34	1.12	1.27	2.01	0.0229
Fruit	1.50	1.92	1.03	1.05	2.27	0.6874
Dairy and alternatives	1.21	1.66	2.63	3.29	2.30	0.0906
Meat and alternatives	0.42	0.58	0.68	0.91	1.24	< .0001
Discretionary foods	2.01	2.42	3.04	2.88	2.94	0.3441
*Vitamin B6*						
Grains	1.47	1.90	1.54	2.04	2.12	0.1229
Vegetables	1.07	1.24	0.97	1.94	1.35	0.2328
Fruit	0.71	1.57	1.13	2.14	2.21	0.0236
Dairy and alternatives	1.55	1.49	2.75	3.54	1.77	0.2735
Meat and alternatives	0.50	0.54	0.61	1.03	1.15	0.0001
Discretionary foods	1.51	2.23	3.93	1.94	3.67	0.0928
*Vitamin B12*						
Grains	1.45	2.05	1.43	1.75	2.39	0.0909
Vegetables	0.85	1.68	1.30	1.52	1.22	0.5947
Fruit	1.51	2.15	1.35	1.38	1.37	0.5217
Dairy and alternatives	1.21	1.15	2.25	3.53	2.97	0.0063
Meat and alternatives	0.54	0.76	0.55	0.85	1.13	0.0112
Discretionary foods	2.42	2.57	2.67	2.20	3.42	0.5057
*Folate*						
Grains	1.33	1.93	1.82	2.18	1.81	0.1974
Vegetables	0.86	1.35	0.92	2.37	1.06	0.1720
Fruit	1.21	1.44	1.77	2.05	1.29	0.6393
Dairy and alternatives	2.14	1.92	1.42	1.97	3.65	0.1742
Meat and alternatives	0.52	0.50	0.75	1.13	0.93	0.0028
Discretionary foods	1.86	1.37	3.56	2.15	4.34	0.0138
*Vitamin B2*						
Grains	2.08	1.67	1.54	1.94	1.83	0.8092
Vegetables	0.92	1.74	1.18	1.61	1.12	0.7908
Fruit	1.67	1.42	2.00	1.62	1.06	0.5320
Dairy and alternatives	1.36	1.58	2.22	2.49	3.44	0.0209
Meat and alternatives	0.55	0.73	0.85	0.93	0.77	0.2294
Discretionary foods	1.16	3.19	3.19	2.14	3.61	0.1090

*P values are for linear trend across the quintiles. Statistically significant association *p* *<* 0.0197

**Table 6:** Food sources of one-carbon metabolism nutrients among WATCH children

Rank	FFQ food item	Consumers, n (%)	%Total nutrient intake
*Methionine*		2 years (*n=*40)	2 years (*n=*40)
1	Plain milk full-cream	25 (63)	20.08
2	Cheese	38 (95)	10.76
3	Plain milk reduced fat	10 (25)	8.23
4	Yoghurt not frozen	39 (98)	6.96
5	Meat mince	38 (95)	4.78
*Methionine*		3 years (*n=*30)	3 years (*n=*30)
1	Plain milk full cream	23 (77)	20.55
2	Cheese	28(93)	8.29
3	Yoghurt not frozen	28 (93)	6.54
4	Meat mince	29 (97)	5.66
5	Plain milk reduced fat	3 (10)	4.86
*Folate*		2 years (*n=*40)	2 years (*n=*40)
1	Breakfast cereal	39 (98)	25.15
2	Vegemite	32 (80)	12.20
3	Plain milk full cream	25 (63)	7.38
4	Banana	38 (95)	4.84
5	Brown bread, pita, roll, toast	24 (60)	4.35
Folate		3 years (*n=*30)	3 years (*n=*30)
1	Breakfast cereal	26 (87)	22.25
2	Vegemite	28 (93)	16.44
3	Plain milk full cream	23 (77)	6.98
4	Yoghurt not frozen	28 (93)	3.66
5	Banana	28(93)	3.51
*Choline*		2 years (*n=*40)	2 years (*n=*40)
1	Plain milk full cream	25 (63)	17.55
2	Plain milk reduced fat	10 (25)	8.30
3	Cream or sour cream	17 (43)	8.21
4	Eggs	33 (83)	7.26
5	Yoghurt not frozen	39 (98)	5.20
*Choline*		3 years (*n=*30)	3 years (*n=*30)
1	Plain milk full cream	23 (77)	17.87
2	Eggs	25 (83)	7.00
3	Cream or sour cream	16 (53)	6.58
4	Plain milk reduced fat	3 (10)	4.88
5	Yoghurt not frozen	28 (93)	4.86
*Vitamin B2*		2 years (*n=*40)	2 years (*n=*40)
1	Plain milk full cream	25 (63)	22.20
2	Vegemite	32 (80)	21.04
3	Breakfast cereal	39 (98)	12.65
4	Yoghurt not frozen	39 (98)	9.12
5	Plain milk reduced fat	10 (25)	8.43
*Vitamin B2*		3 years (*n=*30)	3 years (*n=*30)
1	Vegemite	28 (93)	27.18
2	Plain milk full cream	23 (77)	20.14
3	Breakfast cereal	26 (87)	10.75
4	Tomato & BBQ sauce	23 (77)	7.71
5	Yoghurt not frozen	28 (93)	7.59
*Vitamin B6*		2 years (*n=*40)	2 years (*n=*40)
1	Plain milk full cream	25 (63)	20.61
2	Banana	38 (95)	14.86
3	Breakfast cereal	39 (98)	8.75
4	Chicken crumbed	32 (80)	3.38
5	Plain milk reduced fat	10 (25)	3.37
*Vitamin B6*		3 years (*n=*30)	3 years (*n=*30)
1	Plain milk full cream	23 (77)	15.03
2	Snack bars	15 (50)	11.25
3	Banana	28(93)	9.18
4	Breakfast cereal	26 (87)	6.51
5	Liver	6 (20)	3.91
*Vitamin B12*		2 years (*n=*40)	2 years (*n=*40)
1	Plain milk full cream	25 (63)	22.20
2	Plain milk reduced fat	10 (25)	21.04
3	Cheese	38 (95)	12.65
4	Meat mince	38 (95)	9.12
5	Liver	3 (8)	8.43
*Vitamin B12*		3 years (*n=*30)	3 years (*n=*30)
1	Liver	6 (20)	29.64
2	Plain milk full cream	23 (77)	29.27
3	Meat mince	29 (97)	6.11
4	Cheese	28(93)	5.46
5	Plain milk reduced fat	3 (10)	4.31

#### Folate

The estimated mean total intake of folate per day was 189 µg (±61 µg), which is 58% above the estimated average requirements (EAR) at two years of age. While at three years of age, the daily intake of folate was 194 µg (±56), which is 62% above the EAR. The main food group sources of folate for the WATCH children in the combined age groups were grains (31%), discretionary foods (24%) and dairy and alternatives (18%) (Table 3). As the quintiles of folate intake at two years of age increased (*p* < 0.0197) the daily mean number of serves consumed of grains, vegetables, dairy and alternatives and meat and alternatives were also greater (Table 4). As the quintiles of folate intake at three years of age increased (*p* < 0.0197), the daily mean number of serves consumed of meat and alternatives and discretionary foods were also greater (Table 5). The top five ranked foods for contributing to folate intake that occurred at both two and three years of age were breakfast cereal, vegemite, milk and bananas (Table 6).

#### Choline

The estimated mean total intake of choline per day was 184 mg (±73 mg), which is 8% below the adequate intake (AI) at two years of age. While at three years of age, the daily intake of choline was 180 mg (±52), which is 10% below the AI. The main food group sources of choline for the WATCH children in the combined age groups were dairy and alternatives (34%), discretionary foods (22%) and meat and alternatives (21%) (Table 3). As the quintiles of choline intake at two years of age increased (p < 0.0197), the daily mean number of serves consumed of grains and dairy and alternatives were also greater (Table 4). As the quintiles of choline intake at three years of age increased (*p* < 0.0001), the daily mean number of daily serves of meat and alternatives were also greater (Table 5). The top five ranked foods for contributing to choline intake that occurred at both two and three years of age were milk, cream, eggs and yoghurt (Table 6).

#### Vitamin B2

The estimated mean total intake of vitamin B2 per day was 2 mg (±0.8), which is 413% above the EAR at two years of age. While at three years of age, the daily intake of vitamin B2 was 2 mg (±1.1), which is 475% above the EAR. The main food group sources of vitamin B2 for the WATCH children in the combined age groups were dairy and alternatives (38%), discretionary foods (33%) and grains (15%) (Table 3). As the quintiles of vitamin B2 intake at two years of age increased (p < 0.0197), the daily mean number of serves consumed of grains and dairy and alternatives were also greater (Table 4). As the quintiles of vitamin B2 intake at three years of age increased (*p* < 0.0197), the daily mean number of serves consumed of grains and dairy and alternatives were also greater (Table 5). The top five foods for contributing to the intake of vitamin B2 that occurred at both two and three years of age were vegemite, milk, breakfast cereals and yoghurt (Table 6).

#### Vitamin B6

The estimated mean total intake of vitamin B6 per day was 0.8 mg ( ± 0.2), which is 50% above the EAR at two years of age. While at three years of age, the daily intake of vitamin B6 was 1 mg (±1.5), which is 175% above the EAR. The main food group sources of vitamin B6 intake in the WATCH children in the combined age groups were dairy and alternatives (26%), discretionary foods (20%) and fruit (excluding juice) (18%) (Table 3). As the quintiles of vitamin B6 intake at two years of age increased (*p* = 0.0001), the daily mean number of serves consumed of grains, dairy and alternatives and meat and alternatives were also greater (Table 4). As the quintiles of vitamin B6 intake at 3 years of age increased (*p* < 0.0197), the daily mean number of serves consumed of meat and alternatives were also greater (Table 5). The top five ranked foods for contributing to vitamin B6 intake that occurred at both two and three years of age were milk, bananas and breakfast cereal (Table 6).

#### Vitamin B12

The estimated mean total intake of vitamin B12 per day was 3 µg (±1.7 µg), which is 384% above the EAR at two years of age. While at three years of age, the daily intake of vitamin B12 was 5 µg (±4.3 µg), which is 561% above the EAR. The main food group sources of vitamin B12 for the WATCH children in the combined age groups were dairy and alternatives (61%), meat and alternatives (31%) and discretionary foods (6%) (Table 3). As quintiles of vitamin B12 intake at two years of age increased (*p* < 0.0001), the daily mean number of serves consumed of dairy and alternatives were also greater (Table 4). As the quintiles of vitamin B12 intake at three years of age increased (*p* < 0.0197), the daily mean number of serves consumed of dairy and alternatives and meat and alternatives were also greater (Table 5). The top five ranked foods for contributing to vitamin B12 intake that occurred at both two and three years of age were milk, meat mince, cheese and liver (Table 6). However, liver is a highly concentrated source of micronutrients and only nine children (15%) consumed this food, therefore this finding is likely to be distorted by outliers in the WATCH cohort.

## Discussion

Accumulating evidence suggests that early-life nutrition can significantly impact on the epigenome, influencing phenotype, and disease susceptibility across the lifespan [58–61]. Despite this evidence, few studies have analysed postnatal dietary patterns of a cohort in relation to one-carbon metabolism and DNA methylation. Furthermore, previous studies have mainly focussed on the analysis of dietary patterns in a cohort related to the intake of a single one-carbon metabolism nutrient during childhood [62, 63]. To our knowledge the current study is the first to examine the intake of foods and multiple nutrients related to one-carbon metabolism. In addition, the current study quantified the intake of one-carbon metabolism nutrients using detailed dietary information derived from FFQs, which included serving sizes specific to children aged two to three years. The current study is an important contribution for understanding whether specific dietary patterns prevent or reduce aberrant DNA methylations and phenotypes, thus promoting cognitive development, as well as prevent chronic diseases across the lifespan.

### Consumption of the AGHE food groups

The current study found that at least 60% of children consumed the recommended number of serves for fruit and dairy and alternatives, while less than 20% consumed the recommended number of serves for grains, vegetables and meat and alternatives. Similarly, the Melbourne Infant Feeding, Activity, and Nutrition Trial found that less than 25% of children (*n* *=* 244) at age 3.5 years, consumed the recommended number of serves of grains and vegetables [64]. In addition, evidence from other Australian cohorts has clearly demonstrated that young children consume insufficient serves of meat and alternatives as specified by the AGHE [65–67]. It is speculated that parents lack food preparation and cooking skills to enable them to provide meat options that suits their child’s developmental stage [68, 69]. Consequently, children may develop a preference for meats that are high in saturated fat and sodium including sausages, luncheon meats and crumbed meats [65, 69].

The current study also found that >75% of children exceeded the AGHE recommended number of serves for discretionary foods. Furthermore, the consumption of discretionary foods was found to be ~35% higher for children three years of age compared to two years of age. Secondary analysis of the National Nutrition and Physical Activity Survey (2011–2012) also reported that the consumption of discretionary foods exceeded the AGHE recommendations, increasing from three serves at 2–3 years to six serves at 17–18 years [70]. The excessive consumption of discretionary foods by the children in the WATCH cohort most likely displaced their consumption from items within the five core food groups, especially grains, vegetables, meat and alternatives.

### Food sources of the intake of one-carbon metabolism nutrients

In the combined age groups, the WATCH childrens’ intake of folate, vitamins B2, B6 and B12 on average exceeded the EARs, while the intake of choline was 9% below the AI. The WATCH childrens’ intake of vitamin B2 was 22% higher on average than the mean daily intake reported in the Australian Health Survey (2011–2012) in children aged 2–3 years [71]. In addition, the WATCH children’s intake of vitamin B12 was also 8% higher than the national daily mean intake reported in children aged 2–3 years [71]. The WATCH cohort consumed on average 0.4 serves more of dairy and alternatives compared to the 2–3 year old children included in the Australian Health Survey, which may explain the differences in the intake of vitamin B2 and B12 [72]. Consistent with the findings of current study, a cohort of Romanian children aged 4–6 years (*n* *=* 71) also found they had inadequate intakes of choline, which was 14% below the AI [62].

In the WATCH cohort, the consumption of dairy and alternatives was the highest contributor to the intake of all one-carbon metabolism nutrients, except for folate. Furthermore, full-cream milk was ranked in the top three foods for contributing to the intake of all one-carbon metabolism nutrients. These data confirm that the intake of one-carbon metabolism nutrients in this age group is significantly influenced by their milk consumption. However, excessive consumption of dairy and alternatives was associated with lower intakes of other food groups, including grains, vegetables and meat and alternatives, which could impact on variety of foods consumed, and hence nutrients intakes [73]. Webb et al. [67] also reported that large amounts of milk (35% of energy intake) impacted on the consumption of fruit, vegetables and meats in Australian children aged 16–24 months. These findings highlight the need to educate parents about feeding their children appropriate, but not excessive amounts of milk to ensure a wider variety of food groups are consumed and nutritional requirements for one-carbon metabolism are met.

The food group, meat and alternatives, was the second or third highest contributor to the intake of most one-carbon metabolism nutrients (methionine, choline, vitamin B6 and B12). Meat mince, eggs and liver were the highest ranked foods for the intake of methionine, choline, vitamin B6 and B12. Prelicz et al. [62] and Vennemann et al. [63] also confirmed that meat and alternatives are a major contributor to the intake of choline in this age group. However, the WATCH children did not consume the recommended number of serves of meat and alternatives (0.3 vs.1.0 serving), which may explain why most children did not meet the AI for choline. Prelicz et al., [62] found that an adequate daily intake of choline was associated with the consumption of at least one egg per three day period (*p* < 0.05), and at least one portion of meat (90 g) per day (*p* < 0.05), as well as a minimum of two portions (500 mL) of milk or dairy products (500 mL yoghurt or 60 g cheese) per day[62]. This finding suggests that parents require further education and skills in relation to preparing meat and alternatives for infants to ensure their choline requirements are met. This is particularly important during transition feeding since an infant’s intake of milk decreases which is a significant source of choline.

The WATCH dietary data was collected from 2009 to 2011 which coincides with the transition from voluntary to mandatory food fortification of folic acid, introduced in 2009 [74, 75]. Furthermore, grains and discretionary foods were the highest contributors to the intake of folate, suggesting that synthetic sources of the nutrient significantly contributed to the children’s intake. The current study did find that higher intakes of vegetables was correlated with a higher ranked intake of folate, however only 9% of the sample consumed adequate serves of vegetables in line with the AGHE recommendations. Synthetic folic acid has a higher bioavailability (ranging from 2–90%) compared to naturally derived sources of folate, with bioavailability influenced by a range of factors related to cellular mechanisms that facilitate absorption and metabolism, as well as genetic polymorphisms [76–79]. Naturally derived plant sources of folate primarily exist in a polyglutamated form and are hydolysed by folate hydrolase prior to absorption in the small intestine. In contrast, synthetic folic acid is monoglutamated and does not require hydrolysis prior to absorption, which may partially explain the greater bioavailability [80]. Therefore, the impact the of synthetic compared to naturally derived folate on one-carbon metabolism may differ and therefore warrants further investigation.

The consumption of discretionary foods was ranked into the top three food groups in terms of contributing to the intake of all one-carbon metabolism nutrients. Cream products, sauces, crumbed chicken and snack bars were ranked in the top five foods for contributing to the intake of choline and vitamins B2 and B6. Although, the consumption of discretionary foods significantly contributed to the child’s intake of one-carbon metabolism nutrients, these foods can be a significant source of saturated fat, added sugars and sodium. However, the AGHE only describes discretionary foods as those ‘high in’ saturated fats, added sugars and sodium without providing a quantitative measure. This food group includes mixed dishes such as hamburgers, hotdogs and pizza which contain ingredients that can be included in the five core food groups. In addition, a healthy version of a hamburger (e.g. lean mince pattie, salad, wholegrain bun) can be prepared that significantly contributes to the nutritional intake of individuals. In contrast, this food group does include single ingredient foods such as cream, which is a high source (i.e. 25%) of saturated fat and should be limited in the diet. Therefore, caution should be taken when interpreting the overall nutritional contribution of this food group.

### Study limitations

The current study has a few limitations which must be acknowledged when interpreting the findings. The sample in the current study was small and included proportionally more children of a higher socioeconomic background. In addition, they were predominately well-nourished and therefore, their dietary patterns may not be representative of the broader population and /or specific sub-groups. Furthermore, it is not known whether the WATCH mothers were following any dietary restrictions (e.g. gluten-free) at the time of completing their child’s FFQ. Therefore, this study was unable to take into account the impact of maternal dietary patterns on the childrens’ dietary intake. The possibility of reporting bias can not be excluded, particularly as the FFQ data were proxy-reported by the mothers of the WATCH children. However, the ACAES has been validated to estimate usual total energy intake in Australian children aged 2–3 years using doubly labelled water and micronutrient intakes (including vitamin B2 and folate) in children greater than five years of age [44, 45] and found to be relatively accurate. However, the ACAES has not been validated for estimating the number of AGHE food group serves consumed and so results should be interpreted with caution. Due to the limited Australian food composition data available, the intake of methionine and choline was quantified using food databases from the US and Canada. Therefore, the use of food composition data from other countries is likely to have reduced the accuracy when estimating child intakes of methionine and choline.

## Conclusion

To our knowledge, this is the first study to analyse dietary patterns related to intakes of multiple one-carbon metabolism nutrients in young children. The current study found that the consumption of dairy and alternatives, discretionary foods and meat and alternatives food groups significantly contributed to their intakes of most one-carbon metabolism nutrients in children aged two and three years. The child intakes of choline were below the AI, which could be due to inadequate number of serves of meat and alternatives. Excessive consumption of dairy and alternatives and discretionary foods may displace child intakes of other food groups including grains, vegetables and meat and alternatives. Further research is warranted to understand how dietary patterns impacts on DNA methylation and human development. This is particularly important during early childhood when significant reconfiguration of the epigenome and brain development occurs.
